# Osteoprotegerin as an Emerging Biomarker of Carotid Artery Stenosis? A Scoping Review with Meta-Analysis

**DOI:** 10.3390/diagnostics15020219

**Published:** 2025-01-19

**Authors:** Jerzy Chudek, Marta Pośpiech, Anna Chudek, Michał Holecki, Monika Puzianowska-Kuźnicka

**Affiliations:** 1 Department of Internal Medicine and Oncological Chemotherapy, Medical Faculty in Katowice, Medical University of Silesia, 40-027 Katowice, Poland; martapospiech@poczta.onet.pl; 2 Health Promotion and Obesity Management Unit, Department of Pathophysiology, Faculty of Medical Sciences in Katowice, Medical University of Silesia, 40-752 Katowice, Poland; anna.m.chudek@gmail.com; 3 Department of Internal, Autoimmune and Metabolic Diseases, School of Medicine, Medical University of Silesia, 40-055 Katowice, Poland; holomed@gmail.com; 4 Department of Human Epigenetics, Mossakowski Medical Research Institute, Polish Academy of Sciences, 02-106 Warsaw, Poland; m.puzianowska@imdik.pan.pl; 5 Department of Geriatrics and Gerontology, Medical Centre of Postgraduate Education, 01-813 Warsaw, Poland

**Keywords:** atherosclerosis, carotid stenosis, stroke, osteoprotegerin

## Abstract

**Objective**: In developed countries, stroke is the fifth cause of death, with a high mortality rate, and with recovery to normal neurological function in one-third of survivors. Atherosclerotic occlusive disease of the extracranial part of the internal carotid artery and related embolic complications are common preventable causes of ischemic stroke (IS), attributable to 7–18% of all first-time cases. Osteoprotegerin (OPG), a soluble member of the tumor necrosis factor receptor (TNFR) superfamily, is considered a modulator of vascular calcification linked to vascular smooth muscle cell proliferation and collagen production in atherosclerotic plaques. Therefore, OPG emerges as a potential biomarker (BM) of calcified carotid plaques and carotid artery stenosis (CAS). **Methods**: We performed a literature search of PubMed on OPG in CAS and atherosclerosis published until 2024. **Results**: Increased levels of serum OPG were reported in both patients with symptomatic and asymptomatic CAS, and higher values were observed in those with unstable atherosclerotic plaques. Notably, increased OPG levels were observed regardless of the location of atherosclerosis, including coronary and other peripheral arteries. In addition, chronic kidney disease, the most significant confounder disturbing the association between vascular damage and circulating OPG levels, decreases the usefulness of OPG as a BM in CAS. **Conclusions**: Osteoprotegerin may be considered an emerging BM of global rather than cerebrovascular atherosclerosis. Its diagnostic significance in identifying patients with asymptomatic CAS and their monitoring is limited.

## 1. Introduction

Atherosclerosis is a chronic inflammatory process of the arterial wall and the leading risk factor of coronary artery disease (CAD) and ischemic stroke (IS) [1]. Obesity, physical inactivity, arterial hypertension, diabetes mellitus, dyslipidemia, tobacco smoking, and alcohol consumption were identified as so-called traditional risk factors for atherosclerosis [2]. Chronic inflammation is involved in all steps of atherosclerotic plaque development [3]. Its formation is triggered by the activation and dysfunction of endothelial cells (ECs), resulting in the release of numerous vasoactive molecules. They stimulate both the inflammatory processes and the recruitment and migration of monocytes into the deeper layers of the arterial wall [4], and promote the proliferation and transformation of vascular smooth muscle cells (VSMCs) into myofibroblasts taking part in arterial wall remodeling [5,6] with the formation of atherosclerotic plaques with progressive calcification [7]. Calcifications are present in more than half of carotid plaques. The rim calcification suggests unstable plaques with inflammatory activity, blood leakage, and intraplaque hemorrhage, while dense, nodular calcifications confer greater mechanical stability [7].

Stratification of the IS risk in asymptomatic subjects with extracranial atherosclerosis is a major challenge for modern angiology and neurology. As many as 10–15% of people in the general population above 55–60 years old have hemodynamically significant (≥ 50%) carotid artery stenosis (CAS) [8]. Destabilization of carotid plaque with its rupture and, finally, with thrombus formation is the cause of 20–25% of IS episodes through the mechanism of the ipsilateral intracranial arteries’ embolization [9,10]. CAS is considered a modifiable IS risk factor [9]; nevertheless, the difficulty in identifying asymptomatic subjects who would benefit from endovascular carotid procedures (including stenting) or endarterectomy makes population-based screening not useful [9]. Randomized trials (the Asymptomatic Carotid Surgery Trial and Asymptomatic Carotid Atherosclerosis Study) failed to find the correlation between CAS severity and the CAS-associated IS risk in cohorts with at least 50% stenosis [9,11]. In addition, the progression of asymptomatic to symptomatic disease is low (0.3–2.0%/year) [9,11]. Of note, approximately 80% of disabling IS occurs without warning signs and symptoms [9]. Further investigation is required to determine whether revascularization procedures should be performed earlier in this group of patients.

According to the National Institutes of Health (NIH), a BM may be considered an objectively measured molecule that indicates physiological or pathogenic processes, or response to a therapeutic intervention [12]. Several circulating BMs have been evaluated for their usefulness in assessing the progression of asymptomatic to symptomatic CAS with the final formation of thrombosis on the ruptured plaque [13,14,15,16,17,18,19]. Potentially, such BMs may be useful in identifying asymptomatic subjects with CAS who would benefit from revascularization procedures [20,21].

Therefore, a BM must acquire numerous evaluation criteria, including proof of concept (various levels in subjects with a specific clinical condition), prospective validation (the outcome prediction), incremental value (improvement in prediction), clinical usefulness (improvement in clinical management), and reasonable cost-effectiveness [22]. Osteoprotegerin (OPG) is one of the most promising BMs involved in the calcification process during atherosclerosis development. The knowledge concerning OPG as a useful BM for CAS screening and prediction of conversion from asymptomatic to symptomatic disease remains unsummarized.

This scoping review aims to summarize studies concerning OPG in the development and progression of carotid atherosclerosis, including its potential value as a BM of CAS.

## 2. Methodology

**Figure 1:**
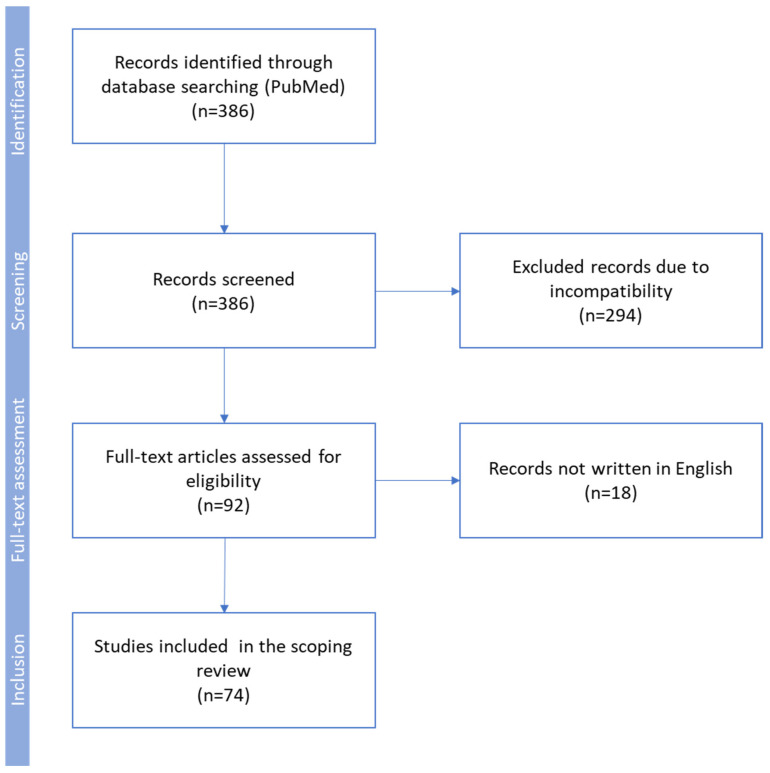
Flow diagram used for identification of studies included in this scoping review. Exclusion criteria: duplicate articles, case reports, editorials, reviews, animal studies not providing important data concerning the pathogenesis of atherosclerosis, and low-quality human studies.

Our review is based on a search of the PubMed database conducted on September 2024, according to the search PRISMA-ScR algorithm: [(OPG) OR (osteoprotegerin)] AND [(carotid stenosis) OR (carotid artery stenosis) OR (CAS) OR (atherosclerosis)]. Initially, 386 records were obtained and independently screened by two reviewers for the content of abstracts. Finally, results from 74 papers concerning osteoprotegerin in human and animal studies, written in English and published since 1997, were included in this systematic review (Figure 1). We excluded duplicate articles, case reports, editorials, and reviews.

We extracted data concerning OPG’s role in the pathophysiology of atherosclerosis, especially the development and progression of carotid atherosclerosis and the role of serum OPG levels as a screening tool for the detection of CAS and predictive significance for conversion from asymptomatic to symptomatic disease. In addition, the papers have been searched for factors decreasing the usefulness of OPG assessment as an emerging BM in clinical practice. Two reviewers assessed the potential risk of bias related to the inclusion criteria and the overall quality of selected papers. The data concerning OPG physiology, its role in the development of atherosclerosis, and its potential usefulness as a BM of carotid artery stenosis were summarized and included in a meta-analysis. The study’s heterogeneity of reported concentrations with various assessment methods limited the meta-analysis to the selected papers applying the same analytical method of OPG quantification. The study was not registered.

### Statistical Analysis

The reported mean levels of OPG with standard deviations and the number of subjects were the source data for the meta-analysis. Heterogeneity among studies was assessed using the I^2^ statistic, with a value greater than 50% indicating significant heterogeneity. Based on the calculated I^2^ value, a random effects model was chosen. Sensitivity analysis was not performed, due to the limited number of studies included in the pooled effect estimation for the endpoint. A *p*-value less than 0.05 was considered statistically significant. All statistical analyses were performed using MedCalc software version 23.0.2 (MedCalc Software Ltd., Ostend, Belgium).

## 3. Results and Discussion

### 3.1. Occurrence of Asymptomatic Carotid Artery Stenosis

The Asymptomatic Carotid Stenosis and Risk of Stroke (ACSRS) Study estimated the 4-year rate of ipsilateral stroke at 5.3% in patients with asymptomatic CAS. Additionally, the higher risk of ipsilateral stroke is in subjects with progressive asymptomatic CAS [23]. According to epidemiological analyses using duplex ultrasound (DUS) as a screening tool, the burden of asymptomatic CAS is even greater. This high burden of disease is explained by the high prevalence of traditional factors predisposed to atherosclerosis, including DM, hypertension, hypercholesterolemia, and cigarette smoking in the aging population [24]. Numerous studies performed worldwide assessed the incidence of severe CAS, ranging from approximately 4.4% to 7.0% [25]. The prevalence of CAS is higher in men than women, and increases with age [26]. Subjects with type 2 DM are 3 times more predisposed to develop asymptomatic CAS than those without DM [27]. Smokers have a 2.68-time greater risk of developing severe CAS than the non-smoker population [28]. In a Japanese study, rural dwellers had a higher prevalence of CAS than city dwellers (9.6% vs. 4.6%), with the difference attributed to long-term hypertension and poor compliance with antihypertensive therapy [29]. Other traditional vascular risk factors were also found to be positively related to an increase in the prevalence of CAS [30,31].

Asymptomatic CAS was more frequently observed in individuals with peripheral artery disease (PAD), as shown in the SMART study (38% of the participants) [32] and in a meta-analysis of 19 studies and 4573 subjects (28% of the included) [33]. Interestingly, classic risk factors for CAS, such as sex, age, smoking, hypercholesterolemia, and hypertension were not predictors for stenosis progression [34,35]. Garvey et al. [36] identified an increase in pulse pressure, a measure of arterial stiffness, and a decrease in HDL cholesterol as the only significant predictors of the progression of carotid plaques [37].

### 3.2. The Physiological Role of Osteoprotegerin

Osteoprotegerin, the tumor necrosis factor (TNF) receptor superfamily member, initially related to osteoclastogenesis inhibition [22], occurs in two forms: a monomer (60 KDa) and a homodimer linked with a disulfide bond (120 KDa), serving as the active form [38]. The molecule plays the role of a soluble ‘decoy receptor’ for receptor activators of nuclear factor kappa B ligand (RANKL) and TNF-related apoptosis-inducing ligand (TRAIL) [22,39,40]. The RANK/RANKL/OPG pathway participates in bone remodeling, osteoclast differentiation, and activation, thus controlling bone turnover [41]. RANK is expressed on the surface of osteoclast precursors, macrophages, monocytes, and dendritic cells [42,43], while RANKL is detected on the membranes of osteoblasts, stromal cells, and T cells [44]. The binding of OPG to TRAIL prevents apoptosis [22,45,46,47]. OPG cysteine-rich domains 1–4 bind to RANKL, while domains 5–6 bind to TRAIL [38,48,49].

Although bone marrow stromal cells remain the primary OPG source, it is also secreted by B-lymphocytes and dendritic cells [50]. OPG production is upregulated by many factors, including calcium ions, 1α, 25-hydroxycholecalciferol, estrogens, bone morphogenetic protein-2 (BMP-2), basic fibroblast growth factor (bFGF), TNFα, transforming growth factor β (TGFβ), platelet-derived growth factor (PDGF), interleukins (IL-1, -6, -7, -11 and IL-18), and angiotensin II (ATII). Additionally, a mechanical stimulus (tensile force) increases OPG synthesis by osteoblasts. Parathyroid hormone (PTH), insulin-like growth factor-I (IGF-I), peroxisome proliferator-activated receptor-γ (PPARγ), prostaglandin E2 (PGE2), glucocorticoids, and immunosuppressants downregulate OPG production [42,51,52,53].

The competitive binding of OPG to RANKL prevents the RANKL–RANK interaction and inhibits osteoclasts, which in turn leads to the protection of bone mass by reducing the intensity of the bone tissue resorption processes [47,54].

### 3.3. The Osteoprotegerin Role in Atherosclerosis

Osteoprotegerin is involved in atherosclerotic disease development; however, studies clarifying its role as a regulator of the disease remain inconsistent. OPG is secreted in atherosclerotic plaques by VSMCs and endothelial cells (ECs) [55], and its secretion is upregulated by pro-inflammatory cytokines such as IL-1, IL-6, and TNFα [56]. The physiological levels of OPG released by ECs and SMCs may prevent calcification of the vessel wall [57]. The level of OPG may, differ depending on the plaque type, with higher concentrations in unstable (symptomatic) carotid atherosclerotic plaques [19]. High concentrations of OPG are related to arterial decalcification and chemoattractant properties of inflammatory cells, especially macrophages, which take part in the release of proteolytic enzymes and can modulate the release of bone enzymes by destroying matrix cells, such as cathepsins. All these mechanisms may promote plaque rupture [19].

Both OPG and RANKL are considered important modulators of atherosclerotic disease [56]. OPG prevents the RANK/RANKL interaction, thereby inhibiting the activation of matrix metalloproteinases (MMPs) in VSMCs [58] and may participate in stabilizing atherosclerosis. Moreover, OPG inhibits VSMCs apoptosis induced by TRAIL [59]. This may explain the OPG cardiovascular protective role [60].

It was shown that OPG deficiency in ApoE-deficient mice leads to advanced plaque progression with expansion in both lesion size and calcification [61]. Moreover, chronic treatment of ApoE-deficient mice with OPG did not affect the size of atherosclerotic lesions, but accelerated smooth muscle accumulation and collagen fiber formation, which led to the stabilization of atherosclerotic plaques by promoting the development of fibrous caps. No alterations in either the systemic or local inflammatory processes were detected [62]. However, Albu et al. showed that increased OPG concentrations may correspond with carotid intima–media thickness (CIMT) among postmenopausal non-diabetic women, and could be considered a cardiovascular risk BM [63]. In a prospective, population-based Tromsø study, Vik et al. indicated that higher serum OPG levels independently predict plaque growth in women; however, OPG was not associated with de novo carotid plaque formation during a 7-year observation [64]. Other authors have shown that serum OPG levels are proportional to increasing arterial stiffness [65] and advanced atherosclerosis [66].

### 3.4. Osteoprotegerin as a Potential Biomarker of Cardiovascular Disorders

Some investigators suggest that OPG protects the cardiovascular system in humans, and may be considered a new BM for atherosclerotic disease [67,68,69]. Higher OPG levels accompany the development of endothelial damage, CAD, PAD, cerebrovascular atherosclerosis [70], aortic aneurysms, and valvular heart diseases [71], as well as heart failure in subjects with past myocardial infarction [72]. In patients with stable CAD, serum OPG values positively correlated with atherosclerosis burden [73]. According to Wajda et al., higher OPG levels were independent and significant predictors of death at admission to the stroke unit [74].

Notably, previous studies have found that serum OPG levels predict the incidence and cardiovascular mortality in CAD patients. In subjects with acute coronary syndrome who developed ST elevation myocardial infarction (STEMI) and qualified for primary percutaneous coronary intervention (PCI), higher OPG concentrations were associated with worse long-term outcomes [75]. In subjects with PAD, its severity correlated with OPG serum levels, and the highest values were associated with ischemic ulcerations [76]. In addition, in symptomatic PAD, increased serum OPG correlated with arterial stiffness measures, such as augmentation index and aortic pulse wave velocity (aPWV) [77], and predicted all-cause mortality [78]. Of note, high arterial stiffness is one of the well-established predictors of poor survival, including isolated hypertension, reduced coronary perfusion pressure, and increased left ventricle (LV) afterload, causing LV remodeling, dysfunction, and heart failure, even in the absence of CAD [79]. High OPG levels were observed in patients with decreased LV ejection fraction and increased LV end-systolic volume. Interestingly, only in men, OPG levels were proportional to LV thickness, mass, and concentricity index [72]. These associations are probably related to the increased arterial stiffness that characterizes patients with increased OPG levels. This statement is supported by the findings in subjects with heart failure with reduced ejection fraction (HFrEF), where serum OPG levels were proportional to arterial stiffness (which correlates with CIMT) [80].

The relationship between serum OPG level and hypertension without concomitant arteriosclerosis remains unclear. ATII participates in the development of hypertension and has an indirect role in the activation of osteoclasts via the RANK/RANKL pathway [75]. The contradictory results of studies concerning OPG levels in hypertensive subjects may be explained by cohort diversity concerning age, ethnicity, kidney function, and the severity of coexisting arteriosclerosis [75,81,82]. In particular, kidney function seems to be a relevant confounding factor for circulating OPG levels, considering the strong inverse association between OPG and glomerular filtration rate [83]. Notably, serum OPG levels in children with idiopathic hypertension are not elevated [84]. This suggests that, in adult hypertensive patients, the serum OPG level is a BM of arterial stiffness and other cardiovascular comorbidities. This hypothesis is supported by results showing higher circulating OPG levels in adults with hypertension and retinopathy, a higher 10-year cardiovascular risk, and at least three damaged target organs (kidneys, heart, and vessels) [85].

Other metabolic disorders should also be considered as confounders that modify the interpretation of increased serum OPG levels, which, for example, were reported in patients with diabetes mellitus (DM), regardless of the type of this disease. A longer duration of DM is associated with increased serum OPG levels [75]. Moreover, higher OPG levels were observed in DM patients with CVD than in those without CVD [75]. These support the hypothesis that increased OPG levels are associated with macroangiopathy, a frequent DM complication closely related to atherosclerosis.

There are inconsistent data regarding serum OPG levels in individuals with metabolic syndrome. Obesity, as the cause of metabolic syndrome, as well as the main hormonal disturbance and insulin resistance, is associated with decreased serum OPG [86,87]. Resistance exercises performed by women with metabolic syndrome, followed by weight loss, increased serum OPG levels, which may be explained not only by the improvement in insulin resistance but also by the effect of resistance exercise on bone mineralization [86]. According to Bergstöm et al., the protective effect of 1-year aerobic training on bone mineralization in postmenopausal women is associated with increased OPG levels [88]. However, other studies reported no change or even a decrease in serum OPG levels after weight loss [87]. Currently, it is difficult to explain these discrepancies.

Generated by obesity, systemic inflammation is mostly related to macrophages that infiltrate and reside in the visceral fat tissue. Obesity-induced low-grade systemic inflammation is considered a link between metabolic syndrome and cardiovascular disease [89]. However, the Framingham Heart Study failed to show an association between OPG, other inflammatory cytokines, and CRP in community-dwelling subjects with metabolic syndrome [90]. Therefore, cytokines other than OPG may be involved in this association.

Summarizing the above-mentioned data, it seems reasonable to state that alterations in serum OPG levels mostly reflect ongoing vascular disease, with some effects on bone metabolism [81,91,92,93,94,95]. Notably, chronic kidney disease frequently observed in older adults is the most important confounder disturbing the association between vascular damage and circulating OPG levels. OPG accumulation in dialysis patients’ circulation has been documented, and the hemodialysis membrane does not remove this protein [96]. Therefore, renal impairment (decreased glomerular filtration rate) should always be considered in the overall assessment of vascular pathology, based on serum OPG levels.

### 3.5. Osteoprotegerin as a Biomarker in Subjects with Carotid Artery Stenosis

Pathophysiological processes within carotid plaques such as lipid accumulation, calcification, inflammation, hypoxia, angiogenesis, proteolysis, and thrombosis are important for the destabilization of carotid plaques. Molecules involved in these processes are released into the circulation.

Many studies tried to determine the usefulness of various serum BMs in predicting stroke risk in patients with carotid plaques and CAS. Potentially, such a non-invasive method of identifying high-risk patients is a promising tool for selecting high-risk patients for carotid surgery [25]. Serum BMs that were presented to be highly correlated with the vulnerability of carotid plaques are mainly markers of inflammation and proteolysis. C-reactive protein (CRP), serum amyloid A (SAA), and IL-6, should be listed among the inflammatory molecules, while MMP-2 and 9, tissue inhibitors of metalloproteinases 1 and 2 (TIMP-1 and TIMP-2), are among the markers of proteolysis. Inhibitors of bone formation are another group of BMs reflecting vascular calcification processes. Osteoprotegerin, osteopontin (OPN), BMP-2, and matrix-carboxyglutamic acid protein (MGP) are the best examples [97,98,99,100]. As mentioned, OPG was shown to modulate vascular calcification [54,68], by inhibiting mineral deposition and osteoclastogenesis [68].

The immunodetection of OPG in carotid plaques indicates its potential role in the atherosclerosis [19,54,101]. Yet, its role in the formation and progression of plaques and response to pharmacological interventions remains unclear [102]. In mice, double inactivation of OPG-/- and apolipoprotein-E-/- (ApoE) accelerated the progression of arteriosclerosis and vessel fibrosis, compared to isolated loss of ApoE [101]. These observations confirm earlier findings in mice with targeted OPG loss/disorders, with increased fibrosis of large arteries and intima–media proliferation [45]. Consistently, the inactivation of OPG expression in transgenic OPG-/- mice was followed by the development of calcified lesions in the arteries of mature mice [103]. In addition, elevated OPG levels in animal models were related to VSMC proliferation (increased count) and enhanced collagen production in plaques, but not to vascularization, the intensity of inflammatory conditions, and plaque size [62]. This was supported by Candido et al. [104], who found that a 12-week exposure to recombinant OPG in ApoE-null mice was correlated with a tiny increase in the total aortic plaque area, but then a significant VSMC extent in plaques with animals receiving the vehicle. There were no differences in the density of macrophage infiltration and collagen fibers in plaques between the two study arms.

### 3.6. Meta Analysis of Studies Assessing Serum Osteoprotegerin in Carotid Artery Stenosis

Eleven studies, summarized in Table 1, assessed serum OPG levels in human subjects with carotid artery plaques and internal carotid artery (ICA) stenosis [102,105,106,107,108,109,110,111,112,113,114].

**Figure 2:**
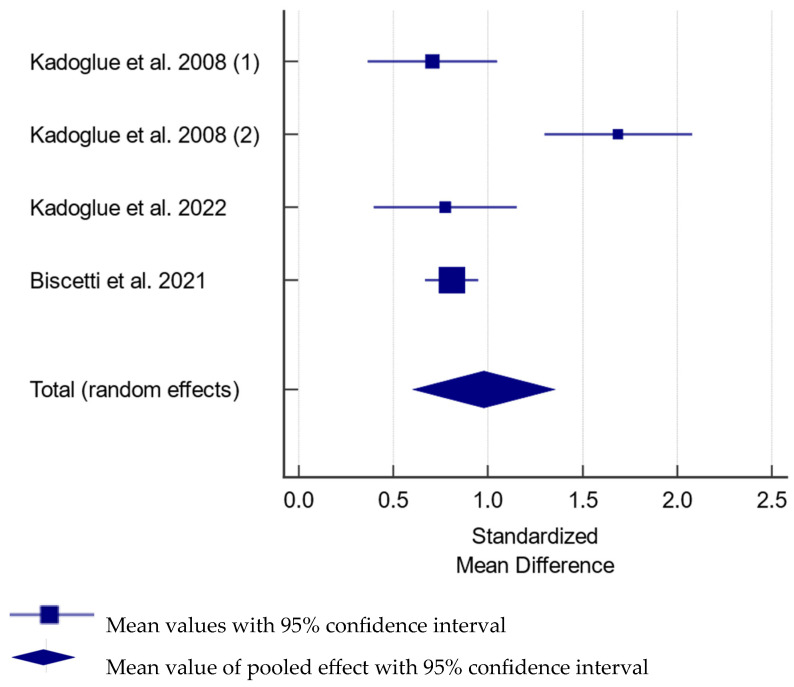
Forest plot showing the difference in serum osteoprotegerin between subjects with and without carotid artery stenosis in studies utilizing reported osteoprotegerin levels in pmol/L [102,105,108,109]. The difference was estimated at 0.968 (95% CI: 0.608–1.328); *p* < 0.001 between the groups for pooled analysis with total random effect due to heterogeneity—I^2^ = 83.2%; N_cases_ = 671, N_controls_ = 666.

All studies using the enzyme-linked immunosorbent assay (ELISA) developed by BioVendor [102,105,108], and established by researchers [109], showed increased OPG levels in subjects with ICA stenosis compared to healthy individuals (Figure 2). The increase in OPG levels in patients with CAS, compared to controls, was estimated at 0.968 (95% CI: 0.608–1.328) pmol/L. A study utilizing ELISA developed by R&D Systems revealed mildly increased OPG levels only in subjects with calcified carotid plaques [112]. Moreover, patients with symptomatic CAS were shown to have increased [102,108] or similar [107,111] OPG levels.

The stenting procedure did not affect the serum OPG levels, precluding considering OPG as the BM of CAS hemodynamics [108].

### 3.7. Osteoprotegerin and Carotid Plaque Vulnerability

The carotid plaque composition affects their vulnerability and cerebrovascular risk, more than the degree of lumen encroachment [115]. Plaques prone to rupture are less calcified, contain large macrophage infiltrates and necrosis areas, and have a thin fibrous cap [115]. Only 15% of carotid plaques contain calcifications [116]. The impact of their calcification on vulnerability and stability is the subject of debate. In coronary circulation, plaque calcifications detected using computed tomography (calcium score) are considered the predictor for coronary episodes [117]. Notwithstanding, more calcifications are found in patients with stable than unstable angina [118]. Of note, calcified plaques in CAS are associated with fewer episodes of transient ischemic attacks and strokes [116]. Therefore, carotid plaque calcification may stabilize them and protect against CAS-related neurologic events.

An open question is whether serum OPG levels may reflect carotid plaque instability (vulnerability). Notably, intraplaque OPG content was positively correlated with carotid plaque stability and circulating OPG levels [111]. Notwithstanding, a single study showed more than twice as high serum OPG levels in diabetic patients with unstable ICA stenosis than in stable disease [109].

The serum concentrations of OPG and other inhibitors of vascular calcification were investigated in subjects with various cardiovascular diseases, and the focus was on plaque instability. Increased OPG concentrations were shown to predict the occurrence of cardiovascular disease [3]. Despite the relevance of plaque calcification with respect to their vulnerability, only a few studies were performed apart from CAD, and their results are inconsistent [68]. Potentially the BMs established for coronary plaque instability may be less useful in detecting the vulnerability of carotid plaques due to differences in the pathophysiological mechanisms of CAS-associated stroke and acute myocardial infarction in CAD. Plaque erosion and thrombotic occlusion are the main mechanisms of myocardial infarction, while plaque rupture and embolization result from advanced unstable plaque in IS [119]. In addition, there are significant differences in the flow rate, shear stress, and histology wall structure between the coronary and carotid arteries, and they may explain the development of morphologically different plaques. High flow rates in the carotid arteries result in a much lower incidence of entire artery occlusion than in the coronary circulation [120]. However, atherosclerotic processes in all vascular beds share many similarities, resulting in plaque vulnerability.

The expectation that OPG as a BM will improve the identification of patients for carotid interventions [37] seems premature. Studies already performed, indicating the increased relative risk of cerebrovascular episodes, do not justify the implementation of OPG and other serum BMs in daily clinical practice.

In addition, the published studies utilizing ELISA show discrepancies concerning measured OPG concentrations in the circulation (Table 1). Most papers assessed the concentration with kits manufactured by BioVendor (Brno, The Czech Republic) and R&D systems (R&D System, Minnesota, MN, USA), with no overlap in the reported concentration ranges after unit conversion. The difference probably results from the lower specificity of antibodies used by R&D systems, followed by higher reported values. This precludes the performance of a meta-analysis, limits the analysis to relative differences reported in individual studies, and postpones the practical application of this BM.

Finally, an association between increased serum OPG levels and cardiovascular diseases was proved [69,121]. Therefore serum OPG cannot specifically identify carotid plaque instability, and should be considered a global indicator of atherosclerosis (Table 2).

## 4. Summary

Carotid atherosclerosis is a significant cause of cerebrovascular morbidity and mortality. The complex mechanisms of the pathogenesis of CAS are not fully understood, and are likely to reflect the interaction of numerous biochemical, immunological, and genetic factors. Undoubtfully, vascular calcification contributes to arteriosclerosis. Increased serum OPG levels correlate with an increased risk of cardiovascular events, including IS. Notwithstanding, knockout mouse models show accelerated atherosclerosis in animals with OPG deficiency. Further studies are necessary to improve our understanding of OPG in the modulation of atherosclerosis development in the carotid arteries, as well as CAS progression and cerebrovascular risk stratification.

**Figure 3:**
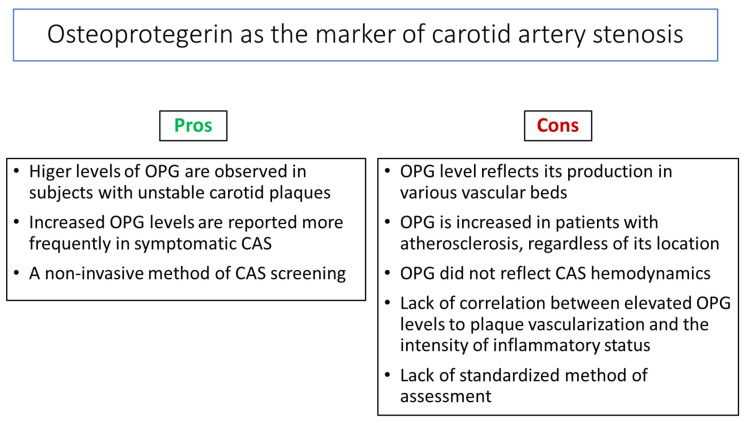
Pros and cons of osteoprotegerin (OPG) assessment as a carotid artery stenosis (CAS) biomarker.

The main challenge is identifying useful BMs to improve the selection of patients with asymptomatic CAS who may benefit from vascular interventions. The current indications for carotid interventions are based on stenosis-related measures, which are usually assessed using Doppler ultrasound or computed tomography angiography and symptomatology. The cost and limited access to these tests decrease their routine use for screening in daily clinical practice. Therefore, developing an easy, non-invasive, and cost-effective way to identify subjects at a high risk of developing CAS complications, including IS, would be advantageous. Nowadays, serum OPG is one of the BMs with unconfirmed potential to identify patients with asymptomatic CAS and those at risk of progression to symptomatic disease (Figure 3).

We cannot exclude that combining OPG with other biomarkers and Doppler ultrasound and other imaging methods in future studies with long follow-up may improve the prediction of CAS progression, leading to the development of risk algorithm calculators.

## 5. Conclusions

Osteoprotegerin may be considered an emerging BM of global, rather than cerebrovascular, atherosclerosis. Its diagnostic significance in identifying patients with asymptomatic CAS and their monitoring is limited.

## Figures and Tables

**Table 1:** The summary of studies assessing osteoprotegerin (OPG) levels in the circulation of human subjects with carotid artery plaques and internal carotid artery (ICA) stenosis.

Cohort Size	Age [Years]	Study Cohort	Comparator	Comparator Age [Years]	OPG Levels in Study Group	OPG in the Comparator Group	Reference
**Osteoprotegerin Quantified Using ELISA (BioVendor)—Concentrations Given in pmol/L**							
114	67.6 ± 6.5	ICA stenosis > 50%	50 age-, sex-, and BMI-matched healthy individuals	65.4 ± 8.9	8.00 ± 3.47 *	5.74 ± 2.39	[105]
97	63.6 ± 9.9	ICA stenosis > 40%	52 healthy controls	60.3 ± 8.8	7.54 ± 2.78 *** Symptomatic 8.15 ± 2.46 Asymptomatic 6.97 ± 2.15	3.39 ± 1.64	[102]
140 (70 + 70)	1. 64.8 ± 7.3 2. 63.2 ± 6.8	ICA stenosis > 30% (<60% for symptomatic or <70% for asymptomatic)	-	-	1. 7.00 ± 2.71 before normal lipid-lowering therapy 2. 6.31 ± 2.56 before aggressive lipid-lowering therapy	-	[106]
113 (46 + 67)	1. 66.8 ± 7.3 2. 64.9 ± 10.4	Symptomatic with >70% ICA stenosis (1.) or asymptomatic with 30–69% ICA stenosis (2.)	-	-	Symptomatic 8.86 ± 3.47 Asymptomatic 9.05 ± 2.65	-	[107]
113	70 ± 9	Asymptomatic with 70–99% ICA stenosis (1.) or symptomatic > 50% ICA stenosis (2.)	38 age-, sex-matched individuals	66 ± 10	8.88 ± 2.74 * Asymptomatic 7.98 ± 2.22 Symptomatic 10.88 ± 3.31 **	6.72 ± 2.88	[108]
**Osteoprotegerin Quantification ELISA (Method Not Specified)—Concentrations Given in pmol/L**							
347 (159 + 188)	1. 72.2 ± 3.3 2. 72.3 ± 3.9	Diabetic patients with unstable (1.) or stable (2.) ICA stenosis	526 diabetic patients without ICA stenosis	71.8 ± 3.8	All 6.86 ± 6.55 *** Unstable 7.65 ± 8.12 *** Stable 3.13 ± 2.23	3.23 ± 2.25	[109]
**Osteoprotegerin Quantified Using ELISA (R&D System)—Concentrations Given in ng/mL**							
91 (54 + 37)	1. 68 ± 6 2. 69 ± 1	Noncalcified (1.) or calcified (2.) carotid plaque	54 age-, sex-, BMI- matched individuals	67 ± 9	Noncalcified 3.21 (median) Calcified 4.11 (median) *	3.20 (median)	[110]
73 (24 + 49)	1. 71 ± 9 2. 69 ± 11	Symptomatic (1.) and asymptomatic (2.) ICA stenosis	-	-	Symptomatic 2.5 ± 1.6 Asymptomatic 3.2 ± 1.5	-	[111]
59	1. 70.5 (68.4–72.7) 2. 68.7 (66.2–71.3)	Echogenic (1.) or echolucent (2.) carotid plaques	41 subjects without carotid plaques	68.1 (66.3–70.0)	Echogenic 1.23 (95%CI: 1.02–1.48) ** Echolucent 1.76 (95%CI: 1.46–2.14)	1.89 (95%CI: 1.60–2.21)	[112]
114 (51 + 63)	1. 73.3 ± 7.4 2. 72.1 ± 6.2	ICA stenosis > 70% with CKD (1.) or without CKD (2.)	-	-	1. 3.19 ± 0.18 2. 3.12 ± 0.11	-	[113]
38 (26 + 12)	1. 72.0 (66.0–77.3) 2. 71.5 (67.3–76.7)	Asymptomatic ICA stenosis > 70% before carotid endarterectomy: treated (1.) or not treated (2.) with statin	-	-	1. 0.17 (0.06–0.32) ^#^ 2. 0.06 (0.06–0.43) ^#^	-	[114]

Data presented as mean values ± standard deviation or (95% confidence interval). Statistical difference in serum OPG in relation to comparator group: * *p* < 0.05; ** *p* < 0.01; *** *p* < 0.001. The conversion rate—1.0 pmol/L = 0.12 ng/mL (OPG molecular weight: 120 kDa). After conversion, there is no overlap in the reported levels. ^#^ Probably a mistake in the assessment or reporting the concentrations.

**Table 2:** Osteoprotegerin and its role in bone and vascular physiology and pathology.

Decoy receptor for RANKL and main inhibitor of osteoclastogenesis and bone resorption [22,41]
Produced by osteoblasts in bones (the main primary source of circulating OPG) [47]
Expressed by VSMCs and endothelial cells (ECs) in atherosclerotic plaques (the second source of circulating OPG) [54,97,98,99,100]
Serum level of OPG is an independent cardiovascular risk factor [3,69], associated with coronary and peripheral artery disease (including CAS) [70], and aortic aneurysms [71]

## Data Availability

Not applicable.
